# Genetic variation and reproductive patterns in wetland mosses suggest efficient initial colonization of disturbed sites

**DOI:** 10.1002/ece3.8255

**Published:** 2021-10-25

**Authors:** Lars Hedenäs, Kristoffer Hylander, Niklas Lönnell, Irene Bisang

**Affiliations:** ^1^ Department of Botany Swedish Museum of Natural History Stockholm Sweden; ^2^ Department of Ecology Environment and Plant Sciences Stockholm University Stockholm Sweden; ^3^ SLU Swedish Species Information Centre Swedish University of Agricultural Sciences Uppsala Sweden

**Keywords:** colonization, disturbance, intraspecific genetic variation, sex expression, sex ratio, succession

## Abstract

To understand colonization processes, it is critical to fully assess the role of dispersal in shaping biogeographical patterns at the gene, individual, population, and community levels. We test two alternative hypotheses (H I and H II) for the colonization of disturbed sites by clonal plants, by analyzing intraspecific genetic variation in one and reproductive traits in two typical fen mosses with separate sexes and intermittent spore dispersal, comparing disturbed, early‐succession (limed) fens and late‐successional rich fens. H I suggests initial colonization of disturbed sites by diverse genotypes of which fewer remain in late‐successional fens and an initially balanced sex ratio that develops into a possibly skewed population sex ratio. H II suggests initial colonization by few genotypes and gradual accumulation of additional genotypes and an initially skewed sex ratio that alters into the species‐specific sex ratio, during succession. Under both scenarios, we expect enhanced sexual reproduction in late‐successional fens due to resource gains and decreased intermate distances when clones expand. We show that the intraspecific genetic diversity, assessed by two molecular markers, in *Scorpidium cossonii* was higher and the genetic variation among sites was smaller in disturbed than late‐successional rich fens. Sex ratio was balanced in *S*. *cossonii* and *Campylium stellatum* in disturbed fens and skewed in *C*. *stellatum* in late‐successional fens, thus supporting H I. In line with our prediction, sex expression incidence was higher in, and sporophytes were confined to, late‐succession compared to disturbed rich fens. Late‐successional *S*. *cossonii* sites had more within‐site patches with two or more genotypes, and both species displayed higher sex expression levels in late‐successional than in disturbed sites. We conclude that diverse genotypes and both sexes disperse efficiently to, and successfully colonize new sites, while patterns of genetic variation and sexual reproduction in late‐successional rich fens are gradually shaped by local conditions and interactions over extended time periods.

## Graphical abstract

We exploit a unique system of recently and large‐scale anthropogenically disturbed habitats and natural rich fens to test two alternative models for recruitment of clonal plants in disturbed sites. Patterns of intraspecific genetic diversity in Scorpidium cossonii (A) and reproductive features in this species and Campylium stellatum (B) show that initial colonization of disturbed sites is by diverse genotypes and a few attain dominance in late‐successional fens and that an initially balanced sex ratio develops into a possibly skewed population sex ratio. We conclude that the genetic variation and sexual reproduction in late‐successional rich fens are gradually shaped by local conditions and interactions over extended time periods.

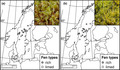

## 1 INTRODUCTION

Since nature is dynamic, organisms need to disperse, either back to places where they recently got extinct or to new areas that have become suitable due to local disturbances or changes in climate or other environmental factors (Clobert et al., 2012; Corlett & Westcott, 2013). The process of dispersal is central to our understanding of biogeographical variability at all hierarchical levels from the gene to the community level (Chust et al., 2016; Clobert et al., 2012). The dispersal ability of organisms depends on traits of their reproductive biology including for plants and other sessile organisms, the traits related to fertilization, and to the formation, release, and transport of their propagules (Johansson et al., 2014; Laenen et al., 2016; Löbel et al., 2018; Muñoz et al., 2004; van Zanten, 1978). However, different types of environmental filtering and local competition are as important as the dispersal per se and interactions thereof, when predicting the composition and dynamics of genes, populations, or communities (Cadotte & Tucker, 2017; Laliberté et al., 2014). Thus, to understand the role of dispersal, there is a need to simultaneously also study colonization processes (Lönnell et al., 2014).

Organisms with small diaspores, such as fungi, ferns, bryophytes, bacteria, or microscopic animals, are often more widely distributed than organisms with large diaspores (Fontaneto, 2011). Nonetheless, the species composition as well as genetic structure of species could still vary across landscapes and regions. For example, many bryophytes with small spores (often <20 µm), which are efficiently dispersed by wind (Wilkinson et al., 2012; Zanatta et al., 2016), have still been found to display clear differences in genetic composition between regions (e.g., Grundmann et al., 2007; Hedenäs, 2009, 2015, 2016, 2019; Shaw, 2000). The detailed analysis of dispersal means and other organismal traits, and the spatial structure of genetic variation can provide fundamental clues to understand different processes during dispersal and colonization (e.g., Aikio et al., 2020; Gjerde et al., 2015; Waters et al., 2013).

It is difficult to find natural systems to study colonization patterns at the landscape scale, the prime example being newly formed volcanic islands (Karadimou et al., 2018; Magnússon et al., 2014). However, Lönnell and Hylander (2018) showed that a unique system of acidic mires that were recently disturbed by regular liming, and that therefore attained a totally different chemical edaphic condition, was perfectly suited to examine colonization processes of vascular plants and bryophytes over large geographic areas. A limed acidic mire is, at least temporarily, transferred into a habitat that in many respects, such as water chemistry and wetness (Löfgren, 2007), resembles a natural rich fen in an early succession stage. Continuous bare surfaces are exposed for possible colonization by typical rich fen species (Rafstedt, 2008).

In this study, we make use of the limed mire system to investigate landscape colonization processes of rich fen habitats. We contrast intraspecific genetic variation in one and reproductive traits in two moss species in early‐succession (i.e., recently disturbed sites, henceforth termed “limed”) and late‐succession (“natural rich”) stages of rich fens. We selected *Scorpidium cossonii* and *Campylium stellatum*, two typical, frequent to dominant mosses in rich fens. Both species are dioicous, show extensive clonal growth, and disperse by spores.

We test two alternative models for recruitment of clonal plants in disturbed sites (Eriksson, 1993). Hypothesis I: Many different genotypes establish on the exposed surfaces at disturbed sites, after which some genotypes gradually disappear and only those best adapted to the local conditions will remain. This hypothesis suggests that the study species will have a higher site‐level genetic diversity and, under conditions of unlimited dispersal, will be genetically more homogeneous across sites of limed fens than across sites of natural rich fens. Provided unlimited dispersal and thus random colonization by spores we anticipate, if this hypothesis is correct, that the two species will exhibit a close‐to‐even sex ratio in the disturbed limed fens and possibly a skewed sex ratio in the late‐successional rich fens (Bisang et al., 2017). Hypothesis II: Initially, only a few genotypes establish in the disturbed fens and rapidly expand vegetatively, followed by a successive recruitment of additional genotypes into the population over extended time. Hypothesis II infers that the species should have a higher genetic diversity, but be genetically more similar across sites, in late‐successional rich fens than in limed fens, because the late‐succession ones have gradually accumulated regionally available genotypes from the spore rain. Under this scenario, sex ratios may be skewed in the limed fens, due to few clones establishing at each disturbed site, and gradually develop to their species‐specific adult population sex ratios in the late‐successional rich fens. Under both hypotheses, we expect sexual reproduction to be enhanced in the late‐successional rich compared to the limed fens after disturbance. First, the plants have had longer time to accumulate resources to devote to the production of sexual structures. Second, the chance for male and female plants to meet increases when clones expand and intermix during habitat succession, which is expected to result in higher fertilization success and thus sporophyte production (Bisang et al., 2004), as long as it is not counteracted by sex‐differential processes during succession. To test these hypotheses, we examined haplotype and nucleotide diversity and distribution of genetic variation in *S*. *cossonii*, and sex expression and sex ratio in *S*. *cossonii* and *C*. *stellatum*, in disturbed limed and natural rich fens, representing early‐ and late‐successional environments. We ask whether within‐ and between‐site patterns of intraspecific genetic variation, sex ratios, and frequencies of sex expression in the study species agree with either of the hypotheses for recruitment of clonal plants in disturbed sites.

## 2 MATERIAL AND METHODS

### 2.1 Study species

**FIGURE 1:**
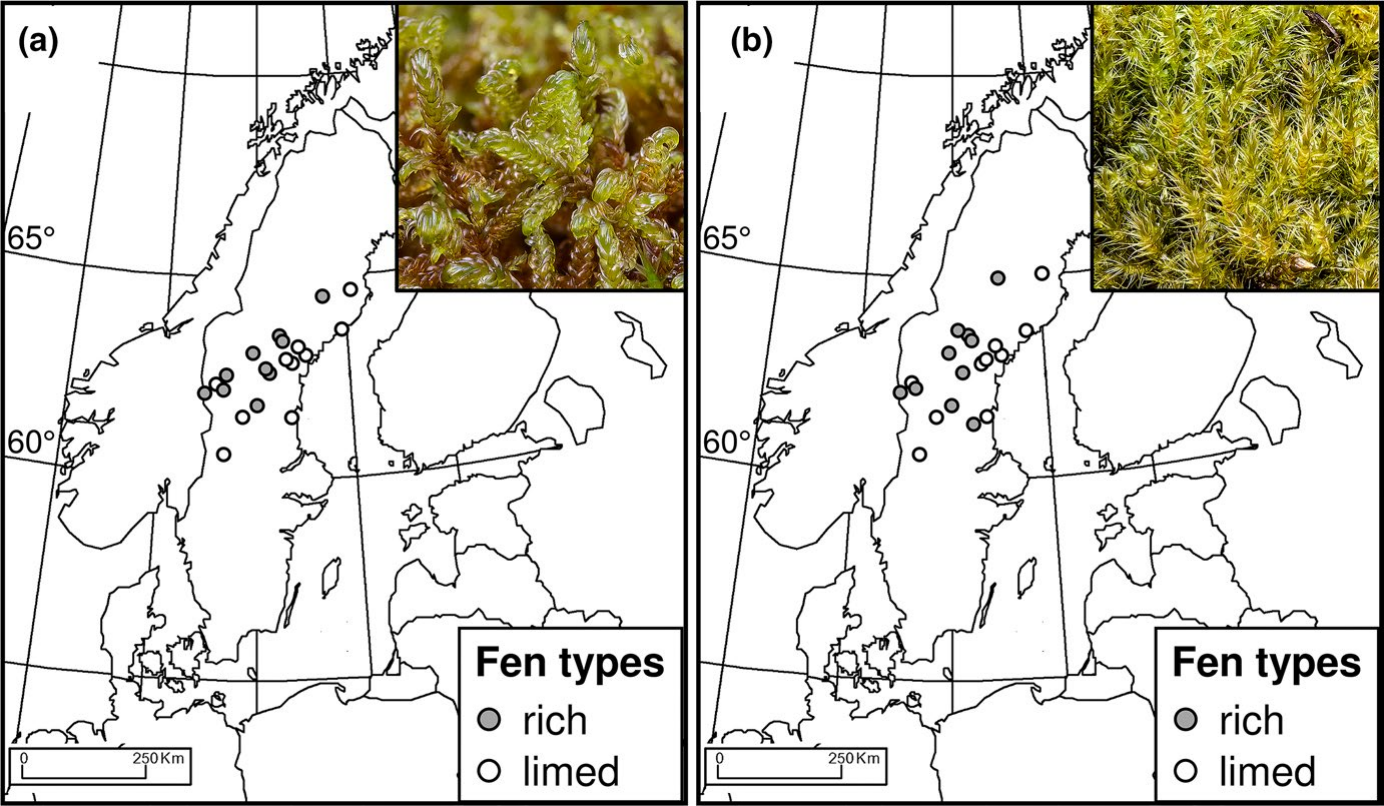
The distribution of the study sites in Sweden, for (a) *Scorpidium cossonii* and (b) *Campylium stellatum*. Photographs by L. Hedenäs

*Scorpidium cossonii* and *Campylium stellatum* are medium‐sized pleurocarpous mosses, members of the families Calliergonaceae and Amblystegiaceae, respectively (Vanderpoorten et al., 2002), and characteristic to and common in base‐rich fens in Scandinavia (Figure 1). The intraspecific genetic variation of *Scorpidium cossonii* is well‐known and geographically structured (Hedenäs, 2009, 2019). Both species have separate sexes (dioicous; Wyatt, 1985). They either expand clonally or, in case of successful sexual reproduction, produce small spores (14–21 and 11–17 µm in diameter) that disperse easily by wind. However, the degree of sexual reproduction varies between the species, and also among populations within each of the species; many shoots or populations do not form sexual structures (Bisang et al., 2019; Bisang & Hedenäs, 2005; McDaniel & Perroud, 2012). Bryophyte sex determination occurs at meiosis when the sex‐determining loci on chromosomes segregate into a spore tetrad that subsequently splits into two male and two female spores, suggesting a balanced progeny sex ratio (Bachtrog et al., 2011; Bisang et al., 2017; Renner et al., 2017). Nevertheless, among reproductive populations and individuals, phenotypic females are usually more common than males (Bisang et al., 2019; Bisang & Hedenäs, 2005; McDaniel & Perroud, 2012). In this study, we investigate phenotypic (functional) sex, that is, sex that is identified based on the presence of reproductive organs. *Scorpidium cossonii* less often forms sexual organs (73% of 331 populations; Bisang et al., 2014) than *C*. *stellatum* (90% of 85 populations; I. Bisang & L. Hedenäs, unpublished data) and exhibits a balanced phenotypic sex ratio in natural vegetation (Bisang et al., 2014). The phenotypic sex ratio of *C*. *stellatum* in natural environments is female‐skewed (I. Bisang & L. Hedenäs, unpublished data). Since the proportion of populations with reproductive structures is higher in *C*. *stellatum* than in *S*. *cossonii*, it likely produces sporophytes more frequently than *S*. *cossonii*.

### 2.2 Geographical setting and study system

Mires cover approximately 5.23 million ha or 11.6% of the total land area of Sweden (Löfroth, 2017). Our study area in the middle‐boreal subzone of Central Sweden (Figure 1) has a continental climate and a bedrock that includes both base‐poor and base‐rich to calcareous regions (Sjörs, 1999). A general description of the mire types in the area is provided by Rydin et al. (1999). Mire size varies strongly in the study area from only a few square meters to hundreds of hectares, forming large wetland complexes (Lönnell & Hylander, 2018). In Central and Northern Sweden, large‐scale liming of acidic mires and other wetlands to mitigate negative effects of acidification in lakes and downstream watercourses of these has been performed since the 1980s (Svenson et al., 1995). Liming of acidic mires kills off the mat‐forming *Sphagnum* spp. and transfers the mires, at least temporarily, into new artificial habitats that resemble natural rich fens in terms of water chemistry and wetness, thereby creating substrates suitable for colonization by rich fen species. In a postglacial perspective, these anthropogenically disturbed habitats became available for colonization by rich fen species very recently. Natural rich fens are sparse in the immediate areas of liming because base‐rich rocks and soil are rare in the region (Sjörs, 1999).

### 2.3 Sampling design

Fieldwork was conducted, and plant material of *S*. *cossonii* and *C*. *stellatum* was sampled, in limed mires (Lönnell and Hylander (2018) and in natural rich fens (N. Lönnell, unpublished data) in Central to Northern Sweden during July–September 2012. At each study site with an ample cover of both or at least one of the study species, the species were sampled from a central 50 × 50 m square in limed fens, or from a corresponding or slightly larger central area in natural rich fens, respectively (Lönnell & Hylander, 2018). At each site, we collected five samples, that is, patches of the study species of ca. 0.25–0.5 dm^2^, distributed across the central sampling area, with a minimum separated distance of 1 meter. The samples were air‐dried and brought to the laboratory.

For this study, we selected 10 limed and 10 natural rich sites for each species in Central Sweden from the available mires, at first‐hand sites where both species co‐occurred in adequate quantities (Table S1; Figure 1). The aim was that the totals of limed and natural sites extended over a comparable geographic area, that the former had been limed at least three times since 1990, and that the latest liming event was conducted <5 years before the sampling. Since not all sites harbored both study species, we sampled a total of 13 limed fens and 14 natural rich fens, the “study sites” (Table S1). Henceforth, we consider the selected samples to represent the study species’ populations at the study sites.

We randomly picked ten individual shoots from the material from each patch to score the presence of male or female sexual structures (sex expression). Out of the 10 *S*. *cossonii* shoots, we randomly picked three shoots for DNA extraction and molecular examination. In a few shoots, the plastid *rpl*16 G2 intron (*rpl*16) belonged to *Bryum pseudotriquetrum* (Hedw.) G. Gaertn., B. Mey. & Scherb. s.l. (Hedenäs et al., 2021). To replace these, we picked and extracted additional shoots and used the first shoot that yielded a complete *S*.* cossonii rpl*16 sequence. For each species, at least one voucher per study site is kept in the herbarium of the Swedish Museum of Natural History, Stockholm (S).

### 2.4 Molecular laboratory work, sequence editing, and analysis of intraspecific genetic variation of *Scorpidium cossonii*

We extracted DNA and generated the nuclear Internal Transcribed Spacers 1 and 2 (ITS) and the plastid *rpl*16 sequences for *S*. *cossonii* as described by Hedenäs (2009), except as follows. The amplified PCR products were purified from excess primers and nucleotides using ExoSap‐ITTM (Applied Biosystems). For all samples, five µl ExoSap‐IT were added to 20 µl PCR product and incubated at 37°C for 30 min followed by an enzyme inactivation step at 80°C for 15 min. We subsequently sent the purified PCR products, together with the same primers used for PCR amplification to Macrogen Europe B.V (Amsterdam, The Netherlands) for single‐stranded sequencing on the Applied Biosystems 3730XL sequencer.

Nucleotide sequence fragments were edited and assembled for each DNA region using PhyDE® 0.9971 (http://www.phyde.de/index.html; accessed 23 March 2020). We aligned the assembled sequences manually in PhyDE®. We identified regions of partially incomplete data in the beginning and end of the sequences and excluded them from subsequent analyses. Gaps were coded using the simple indel coding of Simmons and Ochoterena (2000) in SeqState (Müller, 2005). Gaps provided additional information, and this was included in the analyses. The sequence alignments used in the analyses are available on request.

Internal Transcribed Spacers paralogues are occasionally encountered in bryophytes (e.g., see Hedenäs et al., 2019; Košnar et al., 2012). The ITS chromatograms included in this study did not show “messy” patterns or noise that could suggest paralogy, and the 5.8S gene was invariable among all samples (cf., Feliner & Rosselló, 2007; Shaw et al., 2002). Therefore, the revealed ITS variation was interpreted as being among homologous haplotypes.

We excluded two samples from limed fens because the *rpl*16 sequences of *S*. *cossonii* could not be retrieved. GenBank accession numbers for one specimen per encountered haplotype and haplotype identities for samples are listed in Table S2.

For the analyses of genetic diversity and variation, we consider “site” as sampling unit, from where 15 shoots each were analyzed. The null hypothesis in the following analyses is that there exist no genetic differences between the moss populations from different study sites. We revealed the existing haplotypes based on ITS plus *rpl*16 using the program TCS (Clement et al., 2000). We computed haplotype numbers (*N*_a_), effective number of haplotypes (*N*_e_), haplotype diversity (*H*) (GENALEX 6.501; Peakall & Smouse, 2006, 2012), and nucleotide diversity (*π*) (Arlequin ver. 3.5.1.3; Excoffier & Lischer, 2010) for the populations from each study site. *H* values of 0 indicate a single haplotype and thus potentially a single clone (depending on the genetic markers used), and values of 1 indicate only unique haplotypes and lack of clonality (in analogy with Simpson's D; e.g., Grimsby et al., 2007). We tested for differences in *N*_a_, *N*_e_, *H*, and *π* between fen types with t tests, after checking that the data met the criterion of homogeneity of variance (Levene's test; Brown–Forsythes test; only *N*_a_ was significant at *p* = .0385) and was normally distributed by checking that no outliers were present among the residuals in a preliminary ANOVA. We calculated product–moment correlations among the four diversity parameters. Further, we examined whether haplotype numbers per patch (within the sites), as a proxy for the number of clones, differed between fen types with a nonparametric Mann–Whitney *U* test; patch‐level data did not meet the criterion of homogeneity of variance. Patches were pooled across sites per fen type.

To be able to correctly interpret potential differences between the two fen types, we investigated whether the fen moss populations are currently stable or not with Tajima's *D* test of selective neutrality using Arlequin (Ramírez‐Soriano et al., 2008; Tajima, 1989). Tajima's D suggests that the *S*. *cossonii* populations are stable in all fens of both types except the natural rich fen Korpmyran (Kor; Tajima's D −1.660), where the moss population showed signs of possible increase. For limed fens, it indicates that the initial colonization phase is completed, that is, the population is not further expanding, while populations in the natural rich fens are currently in equilibrium with their habitat.

We investigated patterns of haplotype variation, that is, by partitioning the total variation into the relative amounts within sites, between sites, and between fen types, by analyses of molecular variance (AMOVA; GENALEX). We run AMOVAs for the limed and natural rich fen data separately and for the total data set. We computed average number of pairwise nucleotide differences between populations (Arlequin ver. 3.5.1.3; Excoffier & Lischer, 2010) and estimated genetic variation between populations by means of pairwise Φ_PT_ (an analogue of F_ST_; GENALEX). Genetic variation between populations from different sites based on pairwise Φ_PT_ values and average numbers of pairwise nucleotide differences were visualized with principal coordinate analyses (PCoA; GENALEX). To explore potential isolation‐by‐distance effects, which may obscure habitat effects, we evaluated the relationship between interpopulation Φ_PT,_ based on ITS + *rpl*16 haplotypes_,_ and interpopulation distance by Mantel tests (GENALEX).

### 2.5 Sex expression and sex ratio analyses of *Scorpidium cossonii* and *Campylium stellatum*

We scored the 500 individually sampled shoots per species and habitat type (total = 2000) for sexual structures and categorized those bearing perigonia as phenotypically male (M), those with perichaetia, and if fertilized with sporophytes, as phenotypically female (F), and shoots without sex structures as nonreproductive (NR). Perigonia and perichaetia in pleurocarpous mosses are reduced branches with specialized leaves where male and female gametangia, respectively, are formed and which eventually produce spermatozoids and egg cells. F plus M are termed reproductive shoots. We calculated sex expression (SE) as the proportion of shoots with sexual structures (i.e., reproductive shoots) out of the 10 shoots per patch. We calculated sex ratio (SR) as the number of male shoots divided with total number of shoots with sex organs, that is, a SR > 0.5 indicates male dominance, while SR < 0.5 denotes a female bias. We summed up the occurrence of spore capsules for each species per fen type. We computed mean SE ± standard error and mean SR ± standard error for both natural rich and limed fens for each species across sites, after pooling shoot number per reproductive category (M, F, NR) from five patches per site. We tested whether mean SR per species and fen typed differed from an unbiased SR (0.5) with Pearson's chi‐squared tests. To examine differences in SE and SR between fen type, we used generalized mixed effects models with binomial error distributions and logit link functions. We included fen type as a fixed factor and site as random factor. Lastly, we tested whether number of patches without reproductive shoots, number of unisexual patches, and number of bisexual patches differed between fen type (rich fen or limed fen) for both species using Pearson's chi‐squared tests. Generalized linear models were conducted in the R 4.0.2 environment (R Development Core Team, 2020). For other tests comparing fen habitats, we used STATISTICA 13 (TIBCO‐Software‐Inc., 2017).

## 3 RESULTS

Patterns of genetic variation in *S*. *cossonii* and sex ratios and degrees of sex expression in *S*. *cossonii* and *C*. *stellatum*, both wetland mosses, differed between early‐ and late‐successional environments, that is, limed vs. natural rich fens. These results provided support for the first of the two alternative hypotheses for the colonization of disturbed habitats by clonal plants.

### 3.1 Intraspecific genetic diversity and variation in *Scorpidium cossonii* in relation to fen habitat

**FIGURE 2:**
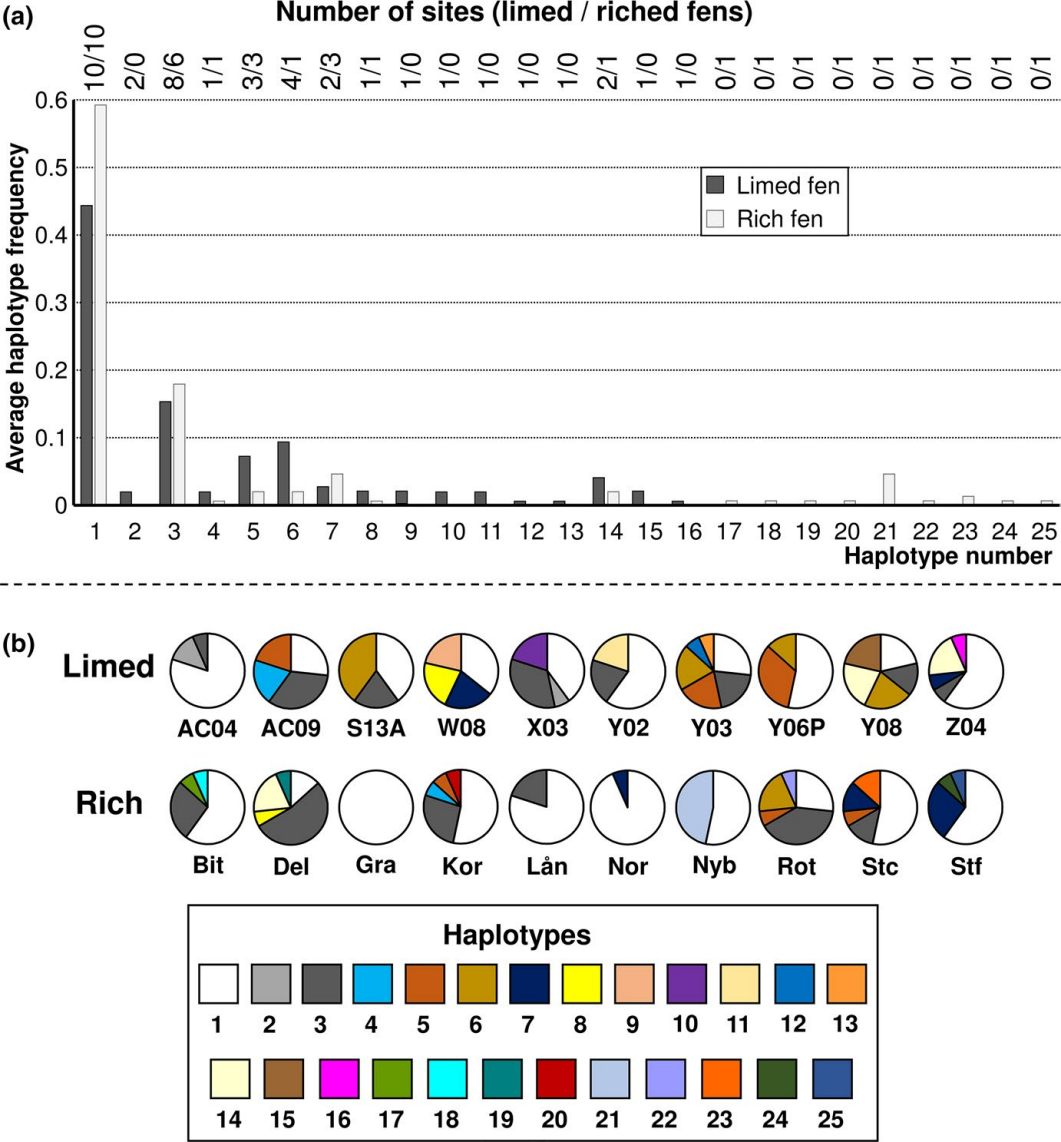
Average haplotype frequency and number of fen sites in which the haplotypes occur (a), and haplotype compositions at the 20 study sites (b), for *Scorpidium cossonii*. For the geographical site location, see Figure 1 and Table S1

**FIGURE 3:**
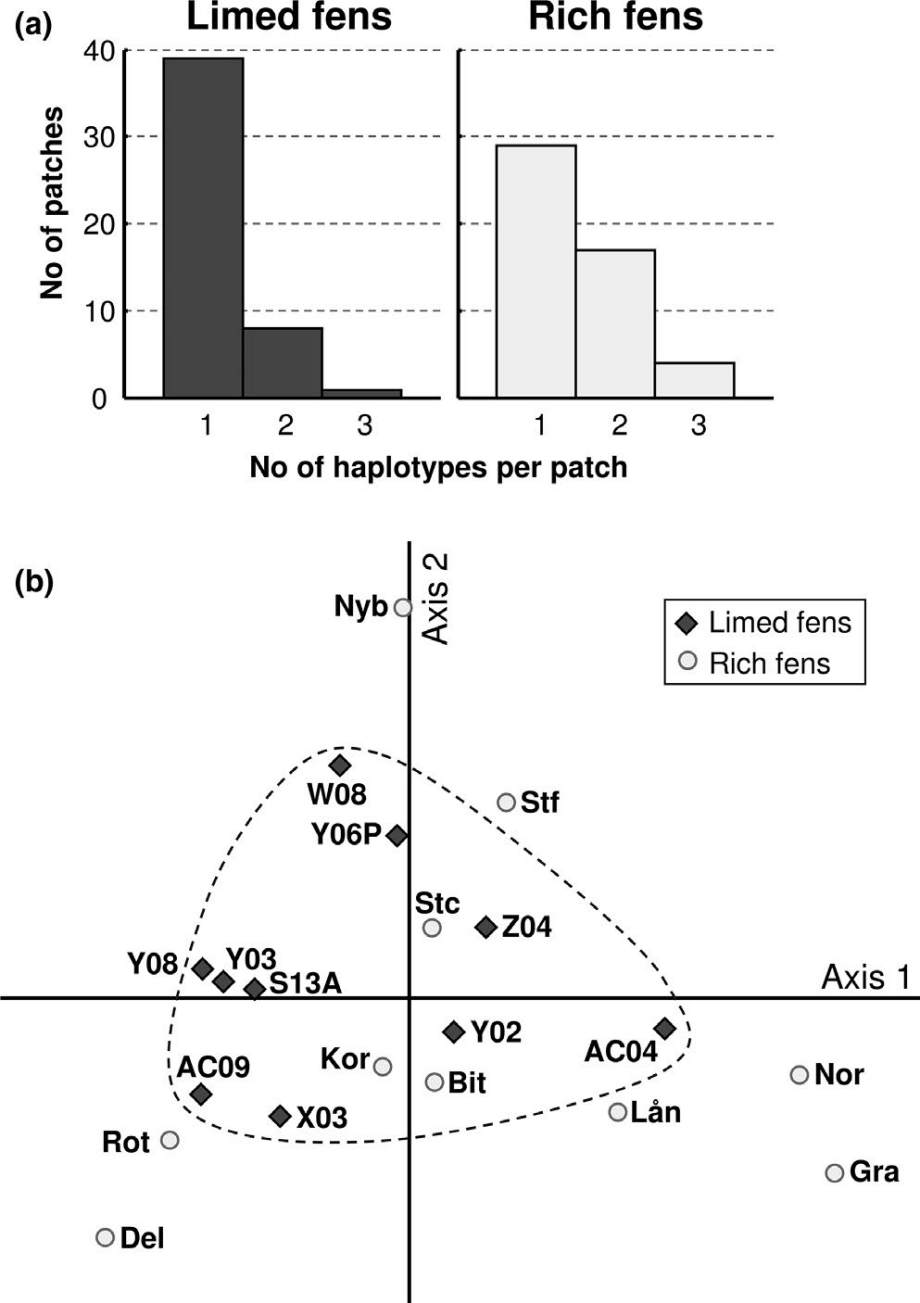
Distribution of haplotype diversity at different scales. (a) Number of haplotypes per patch within site in limed and natural rich fens; *n* = 10 sites per fen type, *n *= 5, patches per site, and *n *= 3 shoots per patch (DNA extraction did not work in 2 shoots from limed fens, and their patches were excluded). Mean haplotype number was lower in limed than in natural rich fens [1.208 (*SE*: 0.066; *n *= 48) vs. 1.500 (*SE*: 0.091; *n *= 50); *p *= .012; Mann–Whitney *U* test; Z adjusted for ties]. (b) PCoA based on pairwise Φ_PT_ values (Table S3A), measuring genetic structure among *Scorpidium cossonii* populations from ten limed (dark grey rhombs) and ten natural rich fen sites (light grey circles). Axis 1, with the eigenvalue 0.186, explains 54.0%, and axis 2, with the eigenvalue 0.048 explains 14.0% of the variation

We did not detect isolation‐by‐distance effects, neither in the entire data set nor among the sites of the respective fen types, and therefore excluded geographical distance from further analysis of the genetic variation (Table 1). The mean diversity, indicated by three diversity parameters *N*_e_, *π*, and *H* (almost significant), was higher in limed than in rich fens (Table 2A). The three diversity parameters were all significantly correlated with each other (results not shown). Average *H* values of 0.646 in the limed fens and 0.472 in the natural rich fens (Table 2A) suggest a mixture of clonal and other recruitment of shoots at the site level, and the difference between the fen types was almost significant. Based on ITS and *rpl*16 combined, we uncovered 25 different haplotypes. Haplotypes 1 and 3 occurred frequently in both fen types, others were uncommon in one (5, 6, 14) or both fen types, and 16 were restricted to a single fen (Figure 2a). The haplotype compositions of the populations showed considerable variation both between study sites within the same fen type and generally between fen types (Figure 2b). At the patch‐within‐site level, mean haplotype number per patch was lower, and more patches carried only a single haplotype, in limed than in natural rich fens (Figure 3a).

**TABLE 1:** Results of Mantel tests for possible isolation‐by‐distance effects based on pairwise Φ_PT_ values (*y*) and geographical distances (*x*) between the *Scorpidium cossonii* populations from ten limed fen and ten natural rich fen sites (9999 permutations)

Populations	*n*	Relation	*R* ^2^	*p*
All populations	190	*y *= 3E−05*x* + 0.1348	.0019	.3425
Limed fens	45	*y* = 4E−05*x* + 0.0882	.0176	.2341
Natural fens	45	*y* = 0.0002*x* + 0.1358	.0324	.2049

Note

*N*, number of comparisons.

**TABLE 2:** (A) Haplotype and nucleotide diversity for *Scorpidium cossonii* populations from ten limed and ten natural rich fen sites in Central Sweden (sampling sites, see Figure 1 and Table S1), based on 25 combined ITS plus *rpl*16 haplotypes. Indel information was included to define haplotypes, but not to calculate nucleotide diversity *n* = number of samples; *N*_a_ = number of haplotypes; *N*_e_ = effective number of haplotypes; *H* = haplotype diversity; *π* = nucleotide diversity [*SD* = standard deviation from Arlequin output (Excoffier & Lischer, 2011)]. Mean values and standard errors (*SE*) across sites of *N*_a_, *N*_e_, *H*, and *π* are indicated for each fen type. Results of t tests to compare mean diversities of limed (*n* = 10) and natural (*n* = 10) rich fens, respectively, are presented at the bottom of A, with statistically significant differences between fen types indicated in bold. (B) Results of AMOVA to partition haplotype variation within and among the ten limed fen sites and the ten natural rich fen sites, and within and among all twenty sites and the two fen types (Total) for *Scorpidium cossonii*, based on ITS and *rpl*16 combined. For limed fens, *n* = 148 (25 haplotypes; Φ = 0.101, *p* = .0001), for natural rich fens *n* = 150 (25 haplotypes; Φ = 0.190, *p* = .0001), and for the total set *n* = 398 (25 haplotypes; Φ = 0.147, *p* = .0001), in all three cases based on 9999 permutations. *df* = degrees of freedom, SS = sums of squares, and MS = mean squared deviations

(A)					
Pop	*n*	*N*_a_	*N*_e_	*H*	[*π* (*SD*)] × 10^3^
Limed fens					
AC04	15	3	1.510	0.338	0.430 (0.408)
AC09	15	4	3.814	0.738	1.137 (0.805)
S13A	15	3	2.778	0.640	0.624 (0.523)
W08	14	4	3.769	0.735	1.176 (0.830)
X03	15	4	3.169	0.684	0.721 (0.577)
Y02	15	3	2.273	0.560	1.123 (0.797)
Y03	15	6	5.000	0.800	1.657 (1.079)
Y06P	15	3	2.419	0.587	0.874 (0.663)
Y08	14	5	4.900	0.796	1.416 (0.958)
Z04	15	5	2.419	0.587	0.998 (0.730)
Mean		4.000	3.205	0.646	1.016
*SE*		(0.333)	(0.364)	(0.044)	(0.117)
Natural rich fens					
Bit	15	4	2.273	0.560	0.637 (0.530)
Del	15	5	2.848	0.649	0.832 (0.639)
Gra	15	1	1.000	0	0 (0)
Kor	15	5	2.711	0.631	0.915 (0.685)
Lån	15	2	1.471	0.320	0.250 (0.292)
Nor	15	2	1.142	0.124	0.097 (0.171)
Nyb	15	2	1.991	0.498	1.164 (0.819)
Rot	15	5	3.571	0.720	1.012 (0.738)
Stc	15	5	2.922	0.658	0.859 (0.654)
Stf	15	4	2.273	0.560	0.541 (0.475)
Mean		3.500	2.220	0.472	0.631
*SE*		(0.500)	(0.263)	(0.077)	(0.126)
Comparing means of limed vs. natural rich fens					
*df*		18	18	18	18
*p*		.416	**.042**	.065	**.038**

(B)					
Source	*df*	SS	MS	Estimated variance	% of total variation
Limed fens					
Among sites	9	8.313	0.924	0.039	10
Within sites	138	47.714	0.346	0.346	90
Total	147	56.027		0.385	100
Natural rich fens					
Among sites	9	10.307	1.145	0.059	19
Within sites	140	35.400	0.253	0.253	81
Total	149	45.707		0.312	100
Total					
Among fen types	1	1.374	1.374	0.002	1
Among sites	18	18.619	1.034	0.049	14
Within sites	278	83.114	0.299	0.299	85
Total	297	103.107		0.351	100

Genetic variation was smaller between sites within limed than between sites within natural rich fens (Table 2B). Accordingly, the genetic differentiation between sites, as measured by pairwise Φ_PT_, was weaker in limed than in natural rich fens (Figure 3b, Table S3A). The first two axes in a PCoA visualizing Φ_PT_ differences explained 68% of the variation (Figure 3b). Also, pairwise nucleotide differences tended to be lower between populations in limed than between populations in natural rich fen sites (Table S3B). The first two axes in a PCoA visualizing nucleotide differences, however, explained only 22% of the variation (not shown). Overall, the variation partitioning (AMOVA) revealed that most of the genetic variation in *S*. *cossonii* (85%) was found within sites and little was referable to between fen types (Table 2B).

### 3.2 Sex ratio and sex expression in *Scorpidium cossonii* and *Campylium stellatum* in relation to fen habitat

**FIGURE 4:**
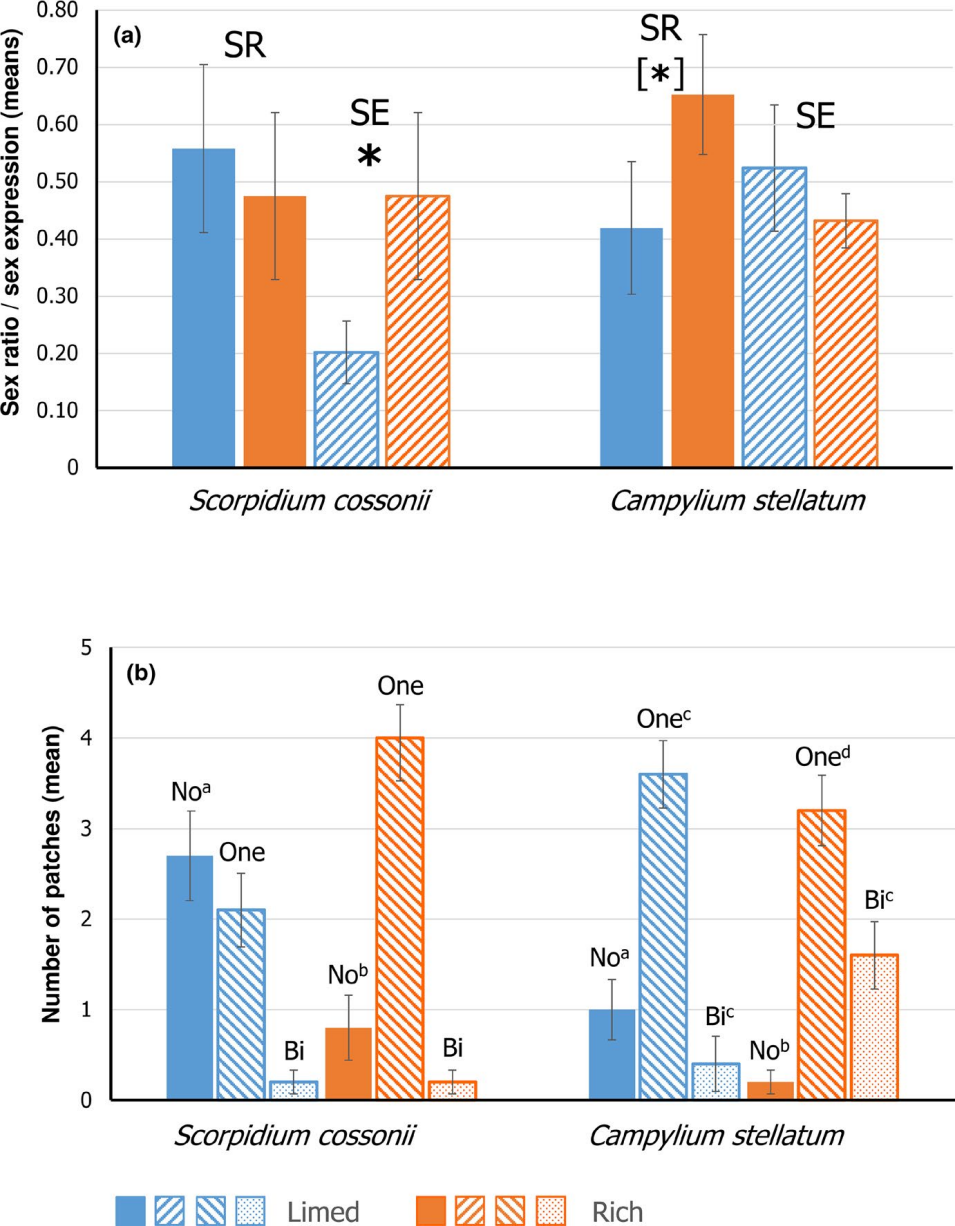
Reproductive traits in *Scorpidium cossonii* and *Campylium stellatum* in limed and natural rich fens in Central Sweden. (A) Means (± *SE*) of phenotypic sex ratio (SR; proportion of male reproductive shoots of total reproductive shoots; solid bars) and sex expression (SE; proportion of shoots with sexual structures; hatched bars) across sites per fen type. *, significant difference at *p *= .003, [*], near‐significant different at *p *= .07, based on generalized mixed effect models (Table 3). (B) Means (± *SE*) of numbers of within‐site patches with different levels of sex expressions across sites per fen type. No, patches without sexual structures on 10 sampled shoots (solid bars); One, patches with shoots with either female or male sexual structures (hatched bars); Bi, patches with both female and male sexual structures (dotted bars); One +Bi is the total number of patches with sexual structures (sex expression). Superscripts a, b indicate that the relative numbers of patches without and with (One +Bi) sex expression differ, and superscripts c, d indicate that the relative numbers of patches with one and with both sexes differ, among fen types

Phenotypic sex ratio variation at the patch‐within‐site level spanned from purely female to purely male in both species and fen types, and the variation was almost as wide at the site level. Mean sex ratio was balanced in limed fens in both species (*S*. *cossonii*: 0.56; *C*.* stellatum*: 0.42; Figure 4A) (Pearson's chi‐squared tests; *X* ^2^ = 2.216; 1.237, *p* = .137; 0.266). In natural rich fens, *S*. *cossonii* exhibited an even sex ratio (0.48; *X* ^2^ = 0.508, *p* = .476), while it was skewed towards males in *C*. *stellatum* (0.65, *X* ^2^ = 40.907, *p* < .001) (Figure 4A). The difference in sex ratios between fen types in *C*. *stellatum*, that is, 0.42 in limed vs. 0.65 in natural rich fens was significant when data within sites were pooled (generalized fixed effect model; estimate [*SE*] 1.0702 [0.1954]; *p* < .001). However, it was only borderline so (*p* = .066) when applying a generalized mixed effect model that takes within‐site variation into account (Table 3A).

**TABLE 3:** Effects of fen type (limed and natural rich fens, representing different succession stages of mire habitats) on reproductive traits in and *Campylium stellatum* (A) and *Scorpidium cossonii* (B) in Central Sweden

Factor	Sex expression			Sex ratio		
	Estimate	*SE*	*p*	Estimate	*SE*	*p*
(A) *Campylium stellatum*						
Fen type	−0.400	0.283	.157	−2.065	1.121	.066
(B) *Scorpidium cossonii*						
Fen type	1.686	0.578	.003	0.946	2.179	.664

Note

Generalized mixed effects models with fen type as fixed and site as random factors. Sex expression, proportion of shoots with sexual structures; sex ratio, proportion of male reproductive shoots of total reproductive shoots. Coefficient estimate (difference against reference fen type; i.e., limed fens), including standard error (*SE*) and *p*‐values (*p*) are presented. See Figure 4 for reproductive traits.

Sex expression at the site level spans from 0% to 54% (limed) and 0% to 100% (natural rich) in *S*.* cossonii*, and 34%–84% and 28%–68% in *C*. *stellatum*, respectively. Mean sex expression was higher in the natural rich compared to limed fens in *S*. *cossonii* (Figure 4A; Table 3B). Sex expression was also higher in natural rich than in limed fens in *C*. *stellatum* when it was analyzed at the patch level (Figure 4B **)**. Despite considerable variation in sex expression within sites, the number of patches without, relative to patches with, sex expression was higher in the limed than in the natural rich fens in both species (Pearson's chi‐squared tests; *S*.* cossonii*, *X* ^2^ = 15.87, *p* < .001; *C*.* stellatum*, *X* ^2^ = 6.06, *p* = .014). Moreover, the number of patches carrying both sexes was higher in natural rich than in limed habitats in *C*. *stellatum* (*X* ^2^ = 6.76, *p* = .009). Sample patches carrying sporophytes were rare in both species, and they were all found in natural habitats (*S*.* cossonii*, 4; *C*.* stellatum*, 5).

## 4 DISCUSSION

We compared genetic diversity and reproductive traits of wetland mosses in a unique system of anthropogenic early‐successional rich fen habitats, recently created by liming, with those in late‐successional natural rich fens. Our findings support hypothesis I for recruitment of clonal plants (Eriksson, 1993), suggesting that a diverse set of genotypes, and both sexes with equal frequencies, colonized disturbed sites initially, also implying that these species easily disperse across several tens of kilometers in few years. We showed that genetic diversity measures in *S*. *cossonii* at the site level, other than haplotype numbers, were higher in limed than in natural rich fens. The limed sites were more similar to each other in genetic composition than were natural rich fens. Sex expression levels in both *S*. *cossonii* and *C*. *stellatum* were higher in the natural rich fens than in the limed fens; sex ratios were even in both species at the limed sites and skewed in *C*. *stellatum* in the natural rich fens. We infer that site‐level genetic diversity and possibly the frequency of one sex decrease with progressing succession, while genetic differentiation among sites increases and more plants reach sexual maturity.

### 4.1 Intraspecific genetic diversity and variation in *Scorpidium cossonii* in relation to fen habitat

We found no isolation‐by‐distance effects on intraspecific genetic composition in *S*. *cossonii*. This agrees with the results of a recent meta‐analysis, where c. 50% of 26 investigated temperate bryophyte species did not show significant IBD signals at ranges of 100–1000 km, that compare to the distances between the sites in this study (Vanderpoorten et al., 2019). Haplotype (and nucleotide) diversity at the site level was higher in limed than in natural rich fens and relative haplotype frequencies differed, while haplotype number per fen was similar in the two fen types (Table 2A). The difference between limed and natural rich fens in genetic diversity resulted from more even relative haplotype frequencies in the former. The higher haplotype diversity, H, in the limed than in the natural rich fens suggests a lower level of clonality and a stronger influence of other modes of shoot recruitment in the limed fens, which represent the early‐successional stage. Further, the limed fen sites displayed less genetic variation between sites and formed a more homogeneous group, than the natural rich fen sites (Table 2B; Figure 2). A higher genetic diversity in disturbed compared to stable habitats was found also in more limited studies of *Sphagnum fallax* and *S*. *capillifolium* (Cronberg, 1996; Daniels, 1985). At the species‐level, a higher number of *Sphagnum* species were recorded where peat had been extracted than in undisturbed peatland communities (Soro et al., 1999). Taken together, these results suggest a large number of recruitment events when a disturbed site is recolonized and a gradual decrease in frequency and sometimes loss of some of the initial genotypes or species, thus supporting hypothesis I (Eriksson, 1993; for vascular plants). In our study system, the *S*. *cossonii* populations were stable, with one possible exception in a natural rich fen (Tajima's D). We therefore infer that the population expansion in the disturbed fens was terminated after at most 30 years since the initial liming events. Further, we suggest that the development between the limed fen environments and the late‐successional natural rich fens, that are several 1000 years old (Sjörs, 1971), was either relatively fast at intermediate ages, or gradual over long temporal scales. At the patch‐within‐site level, more than 80% of the *S*. *cossonii* patches in limed fens included only a single haplotype, reflecting that individual clones after the initial colonization process were still isolated and that at this scale the shoots had not yet grown and intermixed with other clones. In contrast, more than 40% of the *S*. *cossonii* patches in the late‐successional fens contained two or three haplotypes. Thus, even though some clones decrease in frequency or may even disappear with time at a given site, clonal mixing seems to increase within sites.

The variation partitioning disclosed that between‐fen‐type differences accounted for a relatively small amount of the genetic variation. The lack of isolation‐by‐distance effects suggests that diaspore dispersal is not a limiting factor. The limed fens were likely colonized by *S*. *cossonii* from both close and distant sources, which corroborates the findings on species richness by Lönnell and Hylander (2018) from the same sites. The relative amount of genetic variation was highest within sites. *Scorpidium cossonii* frequently produces sporophytes (Bisang et al., 2014) with spores 14–21 µm in diameter (Hedenäs, 1989). Small spores and other particles have theoretically next‐to‐unlimited dispersal potential (Griffin et al., 2002; Ingold, 1984; Wilkinson et al., 2012). In exposed and windy habitats, such as open mires, dispersal may be even more efficient (Lönnell et al., 2012; Sundberg, 2013; Sundberg et al., 2006). Yet, the small but significant variation among limed fen sites (Table 2B) still needs an explanation, which is most likely related to the establishment phase. We noted that also at the limed sites, only a few haplotypes were dominant (Figure 2). Although we did not observe any obvious habitat differentiation between limed sites in the field, environmental filtering at the microhabitat level is probably important during the germination and establishment of individual shoots. Further, chance establishments of rare haplotypes will influence the genetic composition. The higher between‐site differentiation in natural rich fens likely results from a gradual decrease in frequency or even elimination of some haplotypes due to differences between the sites in the late‐successional stage in both abiotic and abiotic factors.

A few haplotypes dominated in most fens, but both the number of fens where an individual haplotype occurred, and the average frequencies of the haplotypes varied. Most haplotypes were rare, and 16 out of 25 haplotypes occurred in only one fen. Similar patterns are common in many organisms (Cronberg et al., 2003; Krebs, 2009; Neubert et al., 2006) and have also been found at the species level in bryophytes of natural rich fens (Vitt et al., 1995).

The lack of dispersal limitations and small genetic variation between sites in *S*. *cossonii* has implications for fen restoration. While source populations must be carefully investigated before re‐introductions in species with large interpopulation differences in genetic setup (Hedenäs, 2016, 2018) and limited dispersal potential, this appears not necessary for Scandinavian *S*. *cossonii* as long as material from the same region is transplanted.

### 4.2 Sex ratio and sex expression in *Scorpidium cossonii* and *Campylium stellatum* in relation to fen habitat

In the early‐successional habitats of limed fens, sex ratios of both species did not differ from 0.5. Given the theoretical expectation and recent experimental results of a balanced offspring sex ratio at spore germination, following the meiotic segregation of sex‐determining loci on bryophyte chromosomes (Bachtrog et al., 2011; Bisang et al., 2017; Renner et al., 2017), this result also supports hypothesis I, with a random colonization by many spores. *Scorpidium cossonii* exhibited an even adult sex ratio irrespective of habitat type, while *C*. *stellatum*, co‐occurring in natural rich fens with *S*.* cossonii*, had a biased sex ratio. A development to a skewed sex ratio during succession supports hypothesis I, suggesting intersexual interactions and limited sexual recruitment (e.g., Field et al., 2013). During the successional vegetation development, changes in small‐scale environmental conditions and competitive relationships may disfavor one sex. Sex differences in interactions with environmental factors, clonal growth, founder effects, historic events, or stochastic factors may lead to biased population sex ratios, as they are commonly observed in bryophytes (Bisang & Hedenäs, 2005; Field et al., 2013). However, mechanisms controlling reproductive traits may also be phylogenetically conserved in some lineages (Bisang et al., 2014). The even population sex ratio of *S*. *cossonii* in natural rich fens in this study is in line with earlier findings for *S*. *cossonii* and other species of the Calliergonaceae in natural habitats. We showed that family affiliation had a stronger effect on adult sex ratio expressions than environmental conditions. For mosses with limited fertilization distances (Bisang et al., 2004), it can be advantageous to retain similar numbers of mates to reduce the risk for spatial sex segregation (Bierzychudek & Eckhart, 1988).

As predicted by both hypotheses, sex expression was higher in natural rich than limed fens in both species. The formation of reproductive structures requires resources that may not be available at early stages of establishment when clones expand predominantly by immature shoots, or when other conditions for reproductive processes are not met (e.g., Karlsson & Méndez, 2005). Rydgren et al. (1998) suggested that the initiation of sex organs depends on shoot size in *H*. *splendens*. The formation of sexual organs, fertilization, and sporophyte development are affected by a variety of physical and chemical properties of the environment (e.g., Chopra, 1984; Hedenäs & Bisang, 2019; Longton, 1990; Sundberg, 2002). Further, only when sexually mature plants grow close to each other, fertilization can occur, given the limited sperm dispersal distances in most mosses (Bisang et al., 2004). Indeed, sporophytes were restricted to the natural rich fen habitats in both species. In agreement, proportions of reproductive shoots, and of shoots with sporophytes, increased with habitat age in the two common terrestrial mosses *H*. *splendens* and *Plagiomnium affine* (Cronberg, 2002; Cronberg et al., 2003). Regardless of the general habitat‐related patterns disclosed in our study, variation in reproductive traits within sites was relatively high in both species.

## 5 CONCLUSIONS

Our results support hypothesis I, namely a large number of initial recruitment events of different genotypes and of both sexes at similar frequencies in the exposed habitats after major disturbance (Eriksson, 1993). *Scorpidium cossonii* spores that colonize the unoccupied limed fens represent the overall genetic variation in the spore rain of the study region, and the sampled shoots represent this spore rain after chance effects and environmental filtering during establishment. This agrees with the generally excellent dispersal abilities of bryophytes (Lönnell & Hylander, 2018, Lönnell et al., 2012, Lönnell et al., 2014, Zanatta et al., 2016; but see Löbel et al., 2006, Virtanen & Oksanen, 2007). The early‐successional stage sites are alike in their genetic makeup and in the overall balanced sex ratios, due to the homogeneous spore rain and their similar recent history and environmental settings. Successively, the fens adjust to local site and climate conditions and the vegetation cover continues to develop. During succession, some genotypes, or possibly one sex, decrease in frequency or are eliminated because they are less fit under the actual environmental conditions or due to stochastic processes. One or a few genotypes will gradually achieve dominance (Figure 2b). In line with this, if the sexes differ in habitat requirements, one sex may prevail if the environment gradually changes during succession (Bisang et al., 2019; Stark et al., 2005). Pleurocarpous mosses, like the study species, expand clonally and shoots of different genotypes may with time intermix at small scales, as shown here at the patch scale. New establishments from locally produced spores or from the spore rain, or small‐scale habitat or temporal variation could potentially counteract dominance of certain haplotypes or one sex in the late‐successional habitats (Soro et al., 1999; Sundberg & Rydin, 2002; Tahvanainen et al., 2003; Tahvanainen & Tuomaala, 2003). While heterogeneity may play a role in late‐successional fens, successful establishment from spores is rare in closed vegetation, in both wetland and terrestrial mosses (Miles & Longton, 2008; Mishler & Newton, 1988; Sundberg & Rydin, 2002). We conclude that, after massive initial recruitment, the extent of genetic diversity and sexual reproduction in wetland mosses in late‐successional natural rich fens are gradually shaped by local conditions and interactions over extended time periods. Finally, the formerly acidic mires that were limed at large scales in Central and Northern Sweden provide an excellent in situ system to study colonization patterns after disturbance at the landscape scale.

## CONFLICT OF INTEREST

None declared.

## AUTHOR CONTRIBUTION

**Lars Hedenäs:** Conceptualization (equal); Data curation (lead); Formal analysis (equal); Funding acquisition (equal); Investigation (equal); Methodology (equal); Visualization (equal); Writing‐original draft (equal). **Kristoffer Hylander:** Conceptualization (equal); Formal analysis (supporting); Funding acquisition (equal); Investigation (supporting); Methodology (equal); Writing‐original draft (supporting). **Niklas Lönnell:** Conceptualization (supporting); Investigation (supporting); Methodology (supporting); Resources (lead); Writing‐original draft (supporting). **Irene Bisang:** Conceptualization (equal); Formal analysis (equal); Funding acquisition (supporting); Investigation (equal); Methodology (equal); Visualization (equal); Writing‐original draft (equal).

## Supporting information

Supplementary Material

## Data Availability

One *Scorpidium cossonii* specimen per encountered haplotype, all from Sweden, was submitted to GenBank (https://www.ncbi.nlm.nih.gov/genbank/), see Table S2.
