# Supplementary material for: Genetic variation and reproductive patterns in wetland mosses suggest efficient initial colonization of disturbed sites
Source: Ecol Evol. 2021 Oct 25;11(22):15846–59. doi: 10.1002/ece3.8255 (PMC8601880; doi:10.1002/ece3.8255)
Supplement: Supplementary file 1 — Supplementary Material [file ECE3-11-15846-s001.pdf]

Supplementary material Appendix for the Ecology & Evolution paper:

Genetic variation and reproductive patterns in wetland mosses suggest efficient initial colonization of disturbed sites

By Lars Hedenäs, Kristoffer Hylander, Niklas Lönnell and Irene Bisang

Supplementary material

Table A1**:** Sample sites (all in Central Sweden) in limed and natural rich fens for *Campylium stellatum* and *Scorpidium cossonii*

Table A2**:** GenBank accession numbers and haplotypes of individual shoots

Table A3: Matrices of pairwise Φ_{PT} values and average number of pairwise nucleotide differences

References

Supplementary material Table A1. Sample sites (all in Central Sweden) in limed and natural rich fens for *Campylium stellatum* (Cam) and *Scorpidium cossonii* (Sco).

Limed fens

Acronym	Locality	Latitude	Longitude	Collecting date
Cam	AC02: Västerbotten, Sörviksmyren	65.11221	20.68326	25 Aug. 2012
Sco	AC04: Västerbotten, Brännvinshålet	64.49185	20.24718	23 Aug. 2012
Sco	AC09: Ångermanland, Abborrtjärn	63.60772	19.51399	10 Aug. 2012
Cam,Sco	S13A: Värmland, Stavsjön	60.28652	13.33414	02 July 2012
Cam,Sco	W08: Dalarna, E of Skidbågbäcken	61.45679	14.15993	22 July 2012
Cam,Sco	X03: Hälsingland, N of Yxberg	61.49538	16.82859	27 July 2012
Cam	Y01: Ångermanland, Rismyran	63.59088	18.85015	09 Aug. 2012
Cam,Sco	Y02: Ångermanland, Stor-Märamyran	63.16257	17.93495	08 Aug. 2012
Cam,Sco	Y03: Ångermanland, N of Lillsjön	63.32228	17.39594	06 Aug. 2012
Cam	Y06B: Medelpad, Stor-Fuskberget	62.72770	17.00283	01 Aug. 2012
Cam,Sco	Y06P: Medelpad, Stentjärnsmyrorna	62.81420	17.12493	31 July 2012
Sco	Y08: Medelpad, at Namnlöstjärn	62.85632	16.80036	30 July 2012
Cam,Sco	Z04: Härjedalen, S of Högvålen	62.24943	12.93835	25 July 2012

Natural rich fens

Acronym	Locality	Latitude	Longitude	Collecting date
Cam,Sco	Bit: Ångermanland, Bitarbäcken	63.42759	16.14468	18 Sept. 2012
Cam	Grb: Härjedalen, Granbacktjärnen	62.15044	12.99352	24 Sept. 2012
Cam	Grö: Jämtland, Gröndalsbodarna	63.69658	15.44245	20 Sept. 2012
Cam	Här: Hälsingland, Härderängena	61.41614	16.14786	12 Sept. 2012
Sco	Del: Härjedalen, Delätesmyren	62.43367	13.48269	24 Sept. 2012
Sco	Gra: Lycksele lappmark, Lake Gravsjön	64.46769	18.68902	28 Sept. 2012
Sco	Kor: Medelpad, Korpmyran	62.62803	15.73180	14 Sept. 2012
Cam,Sco	Lån: Ångermanland, Långflon	63.27884	16.60761	16 Sept. 2012
Sco	Nor: Härjedalen, NW of Nordiåsen	62.11329	13.51217	21 Sept. 2012
Cam,Sco	Nyb: Medelpad, Nybodmyran	62.60156	15.85525	14 Sept. 2012
Cam	Rör: Västerbotten, Rörmyran	64.87126	17.96509	27 Sept. 2012
Cam,Sco	Rot: Hälsingland, S of Rotsjön	61.67142	15.17580	13 Sept. 2012
Cam,Sco	Stc: Dalarna, Stockbäckkölen	61.90135	12.39707	23 Sept. 2012
Cam,Sco	Stf: Jämtland, Storflon	63.10173	14.94194	25 Sept. 2012

Supplementary material Table A2. (**A**) GenBank accession numbers for one *Scorpidium cossonii* specimen per encountered haplotype, all from Sweden (https://www.ncbi.nlm.nih.gov/genbank/). Data format: **Sample No.:** Locality (cf., Table 1); Collection date, *Collector*; GenBank accession numbers for ITS and *rpl*16. '(L)' sequenced from the band that on a long gel moved furthest in a PCR, corresponding with *S. cossonii* (see Hedenäs *et al.*, 2021). (**B**) Haplotypes for the individual shoots per locality (acronym explanations in Supplementary material Table A1).

A.

M1059. AC04: Västerbotten, Brännvinshålet; 23 Aug 2012, *N.Lönnell*; MW284391, MW291578. **M1061.** AC04: Västerbotten, Brännvinshålet; 23 Aug 2012, *N.Lönnell*; MW284392, MW291579. **M1075.** AC09: Västerbotten, Abborrtjärn; 10 Aug 2012, *N.Lönnell*; MW284393, MW291580. **M1077.** AC09: Västerbotten, Abborrtjärn; 10 Aug 2012, *N.Lönnell*; MW284394, MW291581. **M1098.** W08: Dalarna, E of Skidbågbäcken; 22 July 2012, *N.Lönnell*; MW284395, MW291582. **M1104.** W08: Dalarna, E of Skidbågbäcken; 22 July 2012, *N.Lönnell*; MW284396, MW291583. **M1110.** W08: Dalarna, E of Skidbågbäcken; 22 July 2012, *N.Lönnell*; MW284397, MW291584. **M1122.** X03: Hälsingland, N of Yxberg; 27 July 2012, *N.Lönnell*; MW284398, MW291585. **M1134.** Y02: Ångermanland, Stor-Märamyran; 08 Aug 2012, *N.Lönnell*; MW284399, MW291586. **M1144.** Ångermanland, Ed, Lillsjön; 6 Aug 2012, *N.Lönnell*; MT586291, MT590317 (L). **M1179.** Y08: Medelpad, at Namnlöstjärn; 30 July 2012, *N.Lönnell*; MW284400, MW291587. **M1185.** Y08: Medelpad, at Namnlöstjärn; 30 July 2012, *N.Lönnell*; MW284401, MW291588. **M1207.** Bit: Ångermanland, Bitarbäcken; 18 Sept 2012, *N.Lönnell*; MW284402, MW291589. **M1214.** Bit: Ångermanland, Bitarbäcken; 18 Sept 2012, *N.Lönnell*; MW284403, MW291590. **M1224.** Del: Härjedalen, Delätesmyren; 24 Sept 2012, *N.Lönnell*; MW284404, MW291591. **M1259.** Kor: Medelpad, Korpmyran; 14 Sept 2012, *N.Lönnell*; MW284405, MW291592. **M1293.** Nyb: Medelpad, Nybodmyran; 14 Sept 2012, *N.Lönnell*; MW284406, MW291593. **M1309.** Rot: Hälsingland, S of Rotsjön; 13 Sept 2012, *N.Lönnell*; MW284407, MW291594. **M1330.** Stc: Dalarna, Stockbäckkölen; 23 Sept 2012, *N.Lönnell*; MW284408, MW291595. **M1349.** Stf: Jämtland, Storflon; 25 Sept 2012, *N.Lönnell*; MW284409, MW291596. **M1351.** Stf: Jämtland, Storflon; 25 Sept 2012, *N.Lönnell*; MW284410, MW291597. **M1415.** Ångermanland, Ed, Lillsjön; 6 Aug 2012, *N.Lönnell*; MT586299, MT590330 (L). **M1418.** Ångermanland, Ed, Lillsjön; 6 Aug 2012, *N.Lönnell*; MT586302, MT602073 (L). **M1420.** Ångermanland, Ed, Lillsjön; 6 Aug 2012, *N.Lönnell*; MT586304, MT590342. **M1426.** Härjedalen, Tännäs, Högvålen; 25 July 2012, *N.Lönnell*; MT586310, MT590348.

B.

Limed fens: **AC04.** M1053: 1; M1054: 1; M1055: 1; M1056: 1; M1057: 1; M1058: 1; M1059: 2; M1060: 2; M1061: 3; M1062: 1; M1063: 1; M1064: 1; M1065: 1; M1066: 1; M1067: 1. **AC09.** M1068: 3; M1069: 1; M1070: 3; M1071: 1; M1072: 1; M1073: 1; M1074: 4; M1075: 4; M1076: 4; M1077: 5; M1078: 5; M1079: 5; M1080: 3; M1081: 3; M1082: 3. **S13A.** M1083: 6; M1084: 6; M1085: 6; M1086: 6; M1087: 6; M1088: 6; M1089: 1; M1090: 1; M1091: 1; M1092: 3; M1093: 3; M1094: 3; M1095: 1; M1096: 1; M1097: 1. **W08.** M1098: 7; M1099: 7; M1100: 7; M1101: 1; M1102: 1; M1103: 1; M1104: 8; M1105: 8; M1106: 8; M1107: 1; M1109: 1; M1110: 9; M1111: 9; M1112: 9. **X03.** M1113: 3; M1114: 3; M1115: 3; M1116: 3; M1117: 2; M1118: 3; M1119: 1; M1120: 1; M1121: 1; M1122: 10; M1123: 10; M1124: 10; M1125: 1; M1126: 1; M1127: 1. **Y02.** M1128: 1; M1129: 1; M1130: 1; M1131: 1; M1132: 1; M1133: 1; M1134: 11; M1135: 11; M1136: 11; M1137: 3; M1138: 3; M1139: 3; M1140: 1; M1141: 1; M1142: 1. **Y03.** M1415: 12; M1144: 1; M1418: 13; M1146: 6; M1147: 6; M1420: 6; M1149: 5; M1150: 5; M1151: 5; M1422: 1; M1153: 1; M1424: 1; M1155: 3; M1156: 3; M1157: 3. **Y06P.** M1158: 6; M1159: 6; M1160: 5; M1161: 5; M1162: 5; M1163: 5; M1164: 1; M1165: 1; M1166: 1; M1167: 1; M1168: 1; M1169: 5; M1170: 1; M1171: 1; M1172: 1. **Y08.** M1173: 6; M1174: 6; M1175: 6; M1176: 1; M1177: 1; M1178: 1; M1179: 14; M1180: 14; M1181: 14; M1183: 3; M1184: 3; M1185: 15; M1186: 15; M1187: 15. **Z04.** M1188: 1; M1189: 7; M1190: 1; M1191: 1; M1192: 1; M1426: 16; M1194: 14; M1195: 14; M1196: 14; M1197: 1; M1198: 1; M1199: 1; M1200: 1; M1201: 3; M1202: 1.
Natural rich fens: **Bit.** M1203: 3; M1204: 1; M1205: 3; M1206: 3; M1207: 17; M1208: 3; M1209: 1; M1210: 1; M1211: 1; M1212: 1; M1213: 1; M1214: 18; M1215: 1; M1216: 1; M1217: 1. **Del.** M1218: 14; M1219: 3; M1220: 14; M1221: 14; M1222: 3; M1223: 8; M1224: 19; M1225: 1; M1226: 1; M1227: 3; M1228: 3; M1229: 3; M1230: 3; M1231: 3; M1232: 3. **Gra.** M1233: 1; M1234: 1; M1235: 1; M1236: 1; M1237: 1; M1238: 1; M1239: 1; M1240: 1; M1241: 1; M1242: 1; M1243: 1; M1244: 1; M1245: 1; M1246: 1; M1247: 1. **Kor.** M1248: 4; M1249: 3; M1250: 3; M1251: 1; M1252: 1; M1253: 3; M1254: 1; M1255: 1; M1256: 1; M1257: 3; M1258: 5; M1259: 20; M1260: 1; M1261: 1; M1262: 1. **Lån.** M1263: 1; M1264: 1; M1265: 1; M1266: 3; M1267: 3; M1268: 3; M1269: 1; M1270: 1; M1271: 1; M1272: 1; M1273: 1; M1274: 1; M1275: 1; M1276: 1; M1277: 1. **Nor.** M1278: 1; M1279: 1; M1280: 1; M1281: 1; M1282: 1; M1283: 7; M1284: 1; M1285: 1; M1286: 1; M1287: 1; M1288: 1; M1289: 1; M1290: 1; M1291: 1; M1292: 1. **Nyb.** M1293: 21; M1294: 21; M1295: 21; M1296: 1; M1297: 1; M1298: 1; M1299: 1; M1300: 1; M1301: 21; M1302: 1; M1303: 1; M1304: 1; M1305: 21; M1306: 21; M1307: 21. **Rot.** M1308: 1; M1309: 22; M1310: 1; M1311: 6; M1312: 6; M1313: 6; M1314: 3; M1315: 3; M1316: 5; M1317: 3; M1318: 1; M1319: 3; M1320: 3; M1321: 1; M1322: 3. **Stc.** M1323: 1; M1324: 1; M1325: 1; M1326: 3; M1327: 7; M1328: 3; M1329: 7; M1330: 23; M1331: 23; M1332: 1; M1333: 1; M1334: 1; M1335: 1; M1336: 5; M1337: 1. **Stf.** M1338: 7; M1339: 7; M1340: 1; M1341: 1; M1342: 1; M1343: 1; M1344: 1; M1345: 1; M1346: 1; M1347: 7; M1348: 1; M1349: 24; M1350: 7; M1351: 25; M1352: 1.

Supplementary material Table A3. Matrix of pairwise Φ_{PT} values (**A**) and average number of pairwise nucleotide differences (**B**) for ITS and *rpl*16 combined for the populations of *Scorpidium cossonii* from 10 natural rich fen and ten limed fen sites. Values below the diagonal are the Φ_{PT} values (**A**) or average number of pairwise differences (**B**); their corresponding probability values, based on 9999 and 1000 permutations, respectively, are shown above the diagonal. Values in bold indicate significant differences between the populations from two sites ($p < 0.05$); underlined values indicate weak significance ($0.04 < p < 0.05$). Site acronyms of limed fens underlined. For the geographical site locations, see Table 1 and Fig. 2.

**A.**	AC04	AC09	S13A	W08	X03	Y02	Y03	Y06P	Y08	Z04	Bit	Del	Gra	Kor	Lån	Nor	Nyb	Rot	Stc	Stf
AC04	-	**0.003**	**0.010**	**0.004**	**0.022**	0.190	**0.003**	0.041	**0.001**	0.166	0.167	**0.000**	**0.000**	0.115	0.199	0.351	**0.009**	**0.002**	0.112	0.072
AC09	**0.246**	-	**0.035**	**0.009**	0.248	0.051	0.372	0.046	0.042	**0.019**	0.095	0.117	**0.000**	0.353	**0.012**	**0.000**	**0.001**	0.426	0.104	**0.005**
S13A	**0.214**	**0.107**	-	**0.012**	0.090	0.057	0.411	0.064	0.235	**0.035**	0.070	**0.007**	**0.001**	0.125	**0.018**	**0.001**	**0.005**	0.361	0.077	**0.017**
W08	**0.196**	**0.126**	**0.139**	-	**0.019**	**0.026**	**0.021**	**0.020**	**0.014**	0.055	**0.021**	**0.001**	**0.000**	**0.032**	**0.005**	**0.001**	**0.008**	**0.008**	0.150	0.143
X03	**0.156**	0.026	0.083	**0.111**	-	0.185	0.163	**0.019**	0.048	0.078	0.409	0.098	**0.001**	0.367	0.078	**0.001**	**0.009**	0.347	0.209	**0.028**
Y02	0.051	0.101	0.107	**0.116**	0.038	-	0.041	0.051	**0.011**	0.320	0.340	**0.006**	**0.000**	0.382	0.280	**0.016**	**0.031**	0.063	0.431	0.103
Y03	**0.212**	0.000	0.002	**0.089**	0.038	0.089	-	0.249	0.283	**0.027**	0.076	**0.034**	**0.000**	0.218	**0.007**	**0.000**	**0.004**	0.357	0.139	**0.013**
Y06P	**0.136**	0.103	0.104	**0.124**	**0.134**	0.097	0.028	-	**0.013**	0.084	0.047	**0.000**	**0.006**	0.135	**0.028**	**0.006**	**0.012**	**0.021**	0.212	0.049
Y08	**0.260**	0.080	0.036	**0.108**	0.083	**0.137**	0.016	**0.135**	-	0.042	**0.016**	0.046	**0.000**	**0.038**	**0.001**	**0.000**	**0.003**	0.180	**0.026**	**0.003**
Z04	0.039	0.132	**0.120**	0.081	0.077	0.020	**0.101**	0.076	0.094	-	0.322	**0.003**	**0.018**	0.348	0.193	0.047	**0.028**	**0.020**	0.380	0.321
Bit	0.042	0.074	0.090	**0.116**	0.007	0.000	0.074	0.097	**0.127**	0.013	-	**0.017**	**0.000**	0.277	0.296	**0.035**	**0.022**	0.112	0.392	0.130
Del	**0.384**	0.056	**0.178**	**0.208**	0.071	**0.204**	**0.095**	**0.287**	0.090	**0.216**	**0.167**	-	**0.000**	**0.027**	**0.001**	**0.000**	**0.000**	0.252	**0.003**	**0.001**
Gra	**0.095**	**0.461**	**0.429**	**0.395**	**0.389**	**0.250**	**0.416**	**0.327**	**0.465**	**0.214**	**0.250**	**0.599**	-	**0.006**	0.216	**0.000**	**0.005**	**0.000**	**0.006**	**0.000**
Kor	0.066	0.012	0.071	**0.094**	0.000	0.000	0.031	0.059	**0.096**	0.015	0.000	**0.128**	**0.276**	-	0.259	**0.020**	**0.022**	0.203	0.381	0.103
Lån	0.000	**0.213**	**0.196**	**0.210**	0.123	0.018	**0.196**	**0.153**	**0.254**	0.041	0.000	**0.340**	0.143	0.020	-	0.222	**0.008**	**0.015**	0.196	0.077
Nor	0.023	**0.385**	**0.347**	**0.299**	**0.309**	**0.167**	**0.341**	**0.241**	**0.389**	0.125	**0.167**	**0.527**	**0.000**	**0.194**	0.060	-	**0.006**	**0.000**	**0.036**	0.089
Nyb	**0.219**	**0.228**	**0.225**	**0.184**	**0.195**	**0.167**	**0.189**	**0.188**	**0.217**	**0.146**	**0.167**	**0.339**	**0.429**	**0.155**	**0.236**	**0.336**	-	**0.001**	**0.024**	**0.021**
Rot	**0.254**	0.002	0.006	**0.137**	0.010	0.098	0.000	**0.135**	0.034	**0.139**	0.065	0.024	**0.474**	0.031	**0.212**	**0.398**	**0.239**	-	0.077	**0.003**
Stc	0.055	0.065	0.085	0.043	0.031	0.001	0.045	0.038	**0.100**	0.000	0.000	**0.184**	**0.245**	0.000	0.042	**0.151**	**0.135**	0.077	-	0.388
Stf	0.075	**0.172**	**0.154**	0.046	**0.123**	0.062	**0.133**	0.097	**0.165**	0.013	0.062	**0.296**	**0.250**	0.062	0.093	0.132	**0.167**	**0.184**	0.000	-

**B.**	AC04	AC09	S13A	W08	X03	Y02	Y03	Y06P	Y08	Z04	Bit	Del	Gra	Kor	Lån	Nor	Nyb	Rot	Stc	Stf
AC04	-	**0.022**	**0.030**	**0.040**	0.253	0.069	0.097	**0.006**	**0.022**	0.231	0.443	**0.000**	0.227	0.336	0.787	0.221	**0.013**	0.150	0.443	**0.004**
AC09	**1.253**	-	**0.003**	0.508	0.099	0.429	0.125	**0.025**	0.226	0.432	0.098	0.191	**0.002**	0.427	**0.035**	**0.000**	**0.035**	0.211	0.253	**0.001**
S13A	**0.853**	**1.520**	-	0.064	**0.015**	0.011	0.159	**0.010**	0.073	**0.025**	**0.030**	**0.000**	**0.005**	**0.034**	**0.037**	**0.007**	**0.002**	0.303	**0.025**	**0.000**
W08	**1.233**	1.576	1.329	-	0.059	0.183	0.314	**0.012**	0.366	0.221	0.089	**0.008**	**0.000**	0.390	**0.032**	**0.002**	**0.040**	0.509	0.255	**0.003**
X03	0.822	1.373	**1.107**	1.395	-	0.147	0.045	**0.001**	**0.028**	0.128	0.550	**0.003**	**0.002**	0.381	0.185	**0.004**	**0.008**	0.293	0.629	**0.000**
Y02	1.173	1.547	1.440	1.643	1.347	-	0.287	0.059	0.262	0.563	0.313	0.060	**0.016**	0.556	0.148	**0.019**	**0.004**	0.267	0.255	**0.001**
Y03	1.511	2.027	1.627	1.995	1.769	1.947	-	0.357	0.644	0.653	0.072	**0.001**	**0.004**	0.334	0.087	**0.012**	**0.002**	0.375	0.181	**0.001**
Y06P	**1.133**	**1.667**	**1.293**	**1.671**	**1.467**	1.533	1.747	-	0.100	0.237	**0.002**	**0.000**	**0.019**	**0.020**	**0.005**	**0.022**	**0.000**	**0.022**	**0.023**	**0.001**
Y08	**1.405**	1.795	1.500	1.796	**1.595**	1.757	2.081	1.671	-	0.346	0.065	**0.029**	**0.000**	0.223	**0.025**	**0.000**	**0.004**	0.418	0.110	**0.000**
Z04	1.027	1.462	**1.293**	1.529	1.253	1.427	1.800	1.333	1.652	-	0.285	**0.009**	0.094	0.891	0.236	0.106	**0.013**	0.307	0.636	**0.023**
Bit	0.733	1.311	**1.000**	1.319	0.933	1.267	1.667	**1.333**	1.510	1.156	-	**0.001**	**0.039**	0.852	0.682	0.044	**0.011**	0.554	0.557	**0.003**
Del	**1.387**	1.422	**1.653**	**1.614**	**1.373**	1.520	**2.133**	**1.889**	**1.795**	**1.551**	**1.356**	-	**0.000**	**0.005**	**0.001**	**0.000**	**0.000**	**0.010**	**0.000**	**0.000**
Gra	0.333	**1.133**	**0.600**	**1.071**	**0.667**	**1.000**	**1.267**	**0.800**	**1.214**	0.800	**0.533**	**1.400**	-	**0.019**	0.190	1.000	**0.012**	**0.004**	**0.053**	**0.033**
Kor	0.933	1.404	**1.200**	1.433	1.133	1.387	1.787	**1.400**	1.624	1.267	1.044	**1.458**	**0.733**	-	0.443	**0.025**	**0.022**	0.617	0.886	**0.008**
Lån	0.453	**1.120**	**0.720**	**1.100**	0.707	1.040	1.387	**1.000**	**1.271**	0.893	0.600	**1.253**	0.200	0.800	-	0.213	**0.010**	0.181	0.544	**0.010**
Nor	0.400	**1.173**	**0.667**	**1.110**	**0.733**	**1.067**	**1.333**	**0.867**	**1.281**	0.858	0.600	**1.467**	0.067	**0.791**	0.267	-	**0.003**	**0.007**	0.107	0.156
Nyb	**1.547**	**1.849**	**1.813**	**1.871**	**1.693**	**2.027**	**2.480**	**2.200**	**2.281**	**1.889**	**1.622**	**1.991**	**1.400**	**1.760**	**1.413**	**1.404**	-	**0.010**	**0.027**	**0.014**
Rot	1.040	1.520	1.147	1.481	1.213	1.493	1.840	**1.524**	1.652	1.400	1.133	**1.511**	**0.867**	1.307	0.907	**0.933**	**1.893**	-	0.331	**0.000**
Stc	0.893	1.409	1.160	1.424	1.067	1.400	1.773	**1.378**	1.633	1.253	1.022	**1.542**	**0.667**	1.178	0.760	0.716	**1.693**	1.302	-	0.084
Stf	**0.800**	**1.467**	**1.067**	**1.395**	**1.133**	**1.467**	**1.733**	**1.267**	**1.681**	**1.222**	**1.000**	**1.867**	**0.467**	**1.147**	**0.667**	0.489	**1.556**	**1.333**	1.044	-

References to Supplementary material

Hedenäs L, Larsson P, Cronholm B, Bisang I. 2021. Evidence of horizontal gene transfer between land plant plastids has surprising conservation implications. *Annals of Botany* online ahead of print.
